# Supplementary material for: scMuffin: an R package to disentangle solid tumor heterogeneity by single-cell gene expression analysis
Source: BMC Bioinformatics. 2023 Nov 27;24:445. doi: 10.1186/s12859-023-05563-y (PMC10680269; doi:10.1186/s12859-023-05563-y)
Supplement: Supplementary file 1 — Additional file 1. Supplementary methods, Figure S1 and Table S1. [file 12859_2023_5563_MOESM1_ESM.pdf]

Supplementary Material of “scMuffin: an R package to disentangle solid tumor heterogeneity by single-cell gene expression analysis”

Valentina Nale[1], Alice Chiodi[1], Noemi Di Nanni[1], Ingrid Cifola[1], Marco Moscatelli[1], Cinzia Cocola[1], Matteo Gnocchi[1], Eleonora Piscitelli[1], Ada Sula[1], Ileana Zucchi[1], Rolland Reinbold[1], Luciano Milanesi[1], Alessandra Mezzelani[1], Paride Pelucchi[1*+], and Ettore Mosca[1*+]

[1]National Research Council, Institute of Biomedical Technologies, Via Fratelli Cervi 93, 20054, Segrate (Milan), Italy

e-mail addresses:

- ettore.mosca@itb.cnr.it

- paride.pelucchi@itb.cnr.it

[+]Corresponding author

SUPPLEMENTARY METHODS **2**

Single cell data analysis 2

SUPPLEMENTARY FIGURE **3**

Figure S1. Transcriptional complexity, proliferation rate and cell state trajectories (PJ016). 3

SUPPLEMENTARY TABLE **4**

Table S1. Overview of the analysis provided by scMuffin and scCancer. 4

REFERENCES **5**

Supplementary Methods

Single cell data analysis

The filtered genes-by-cells count matrix of each sample was downloaded from the Gene Expression Omnibus (GEO) repository and was processed using the R package Seurat (1). Only cells with at least 200 expressed genes, less than 8’000, 6’000 or 3’000 genes (respectively in samples PJ016, PJ017 and PJ030), less than 30’000, 15’000 and 6’000 total counts (respectively in samples PJ016, PJ017 and PJ030), and less than 10% of mitochondrial genes, were considered. Genes expressed in less than 100 cells were excluded. The resulting matrices were log-normalized using the Seurat “NormalizeData” function. In summary, we obtained:

**Sample (GEO accession)**	**#genes**	**#cells**
PJ016 (GSM2758471)	12’126	2’828
PJ017 (GSM2758472)	3746	642
PJ030 (GSM2758475)	4’948	1’173

Cell clustering was performed with Seurat: principal component analysis (PCA) was run on the 2’000 most variable genes, identified by means of “FindVariableFeatures” (method “vst”); “FindNeighbors” was run on the top 10 PCs; “FindClusters” was run with default parameters. The UMAP coordinates were obtained by means of the Seurat “RunUMAP” function, on the top 10 PCs.

Supplementary figure

Figure S1. Transcriptional complexity, proliferation rate and cell state trajectories (PJ016).

a-c) Distribution of cells according to the first two “diffusion components” (DC): colors indicate (a) Transcriptional Complexity Linear Model Residual (TC-LMR), (b) Transcriptional Complexity - Entropy (TC-H) and (c) proliferation score. **d-e**) Distribution of (d) TC-LMR and (e) TC-H values split by proliferation score.

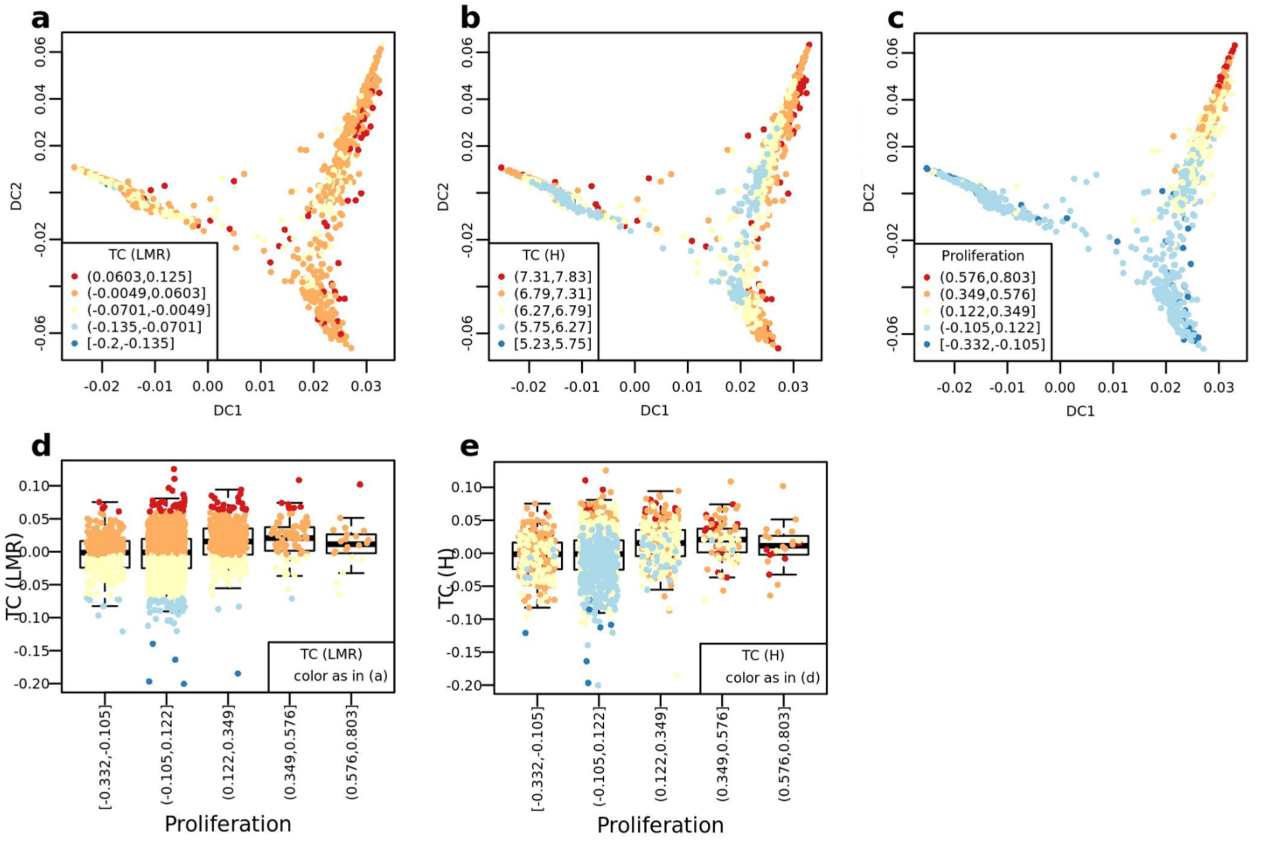

Supplementary table

Table S1. Overview of the analysis provided by scMuffin and scCancer.

**Analysis**	**scMuffin (this work)**	**scCancer** (2)
Quality control, filtering, normalization, cell clustering, dimensionality reduction and cluster marker identification	NA	- Cell statistics (nUMI, nGene)<br>- Gene statistics (mitochondrial genes, ribosomal genes, ambient genes)<br>- Based on Seurat (V3 (1))
Cancer micro-environment cell type classification	NA	- data-driven one-class logistic model
Gene set scoring	- Algorithm by Tirosh *et al*. (3), with support for missing values<br>- Fine-tuning of parameters<br>- Predefined gene sets: CancerSEA, MSigDB, Cell marker, PanglaoDB<br>- Parallel implementation<br>- Cell- and cluster-level gene set scores<br>- Visualization: Heatmap, UMAP	- GSVA and Seurat AddModuleScore() (based on Tirosh *et al.* (3))<br>- Predefined gene sets: MSigDB Hallmarks<br>- Limited control over parameters<br>- Cell-level gene set scores<br>- Visualization: Heatmap
CNV inference	- Based on adjacent gene windows approach by Patel *et al*. (4)<br>- Parallel implementation<br>- CNV region detection<br>- Cell CNV summary score<br>- CNV clusters<br>- Visualizations: CNV Heatmap (with annotation of genes or CNV regions); cluster median CNV profile; cell CNV summary score (per cluster, cell type);	- based on InferCNV algorithm<br>- Cell-level "malignancy" estimation<br>- cell classification (malignant, normal)<br>- Visualization: CNV heatmap, malignancy score over t-SNE, amount of malignant cells per cluster
Stemness	- Transcriptional complexity (TR-Ratio, TR-LMR, TR-H)	- Spearman correlation coefficient between cells' expression and stemness signature (One Class Logistic Regression trained over a stem/progenitor database)
Comparison of multiple cell partitions	- Support multiple cell partitions<br>- Overlap matrix between all-pairs of clusters	NA
Cell cycle/cell proliferation	- Proliferation score (maximum between the gene set scores for G1/S and G2/M)	- gene set scoring (based on Seurat AddModule Score) of cell cycle genes (G2/M and S phase markers)
Expression programs	NA	Based on non-negative matrix factorization
Cluster association analysis	- quantitative features: Cell Set Enrichment Analysis (CSEA)<br>- categorical features: Over Representation analysis (ORA)<br>- Visualization: boxplots, barplot, heatmap	NA
Survival analysis	NA	Survival analysis on gene expression data (patient level) based on marker genes or signatures extracted from SC analysis
Cell interaction	NA	Based on FANTOM5 ligand-receptor interaction and algorithm by Kumar *et al.* (5)

Dataset integration	NA	Various algorithms: "NormalMNN". "Harmony", "NormalMNN", "SeuratMNN", "Raw", "Regression" and "LIGER
Dataset comparison	Assessment of gene set expression across cell clusters from multiple datasets	NA

References

1. Hao Y, Hao S, Andersen-Nissen E, Mauck WM, Zheng S, Butler A, et al. Integrated analysis of multimodal single-cell data. Cell [Internet]. 2021 Jun;184(13):3573-3587.e29. Available from: https://linkinghub.elsevier.com/retrieve/pii/S0092867421005833
2. Guo W, Wang D, Wang S, Shan Y, Liu C, Gu J. scCancer: a package for automated processing of single-cell RNA-seq data in cancer. Brief Bioinform. 2021 May 20;22(3).
3. Tirosh I, Izar B, Prakadan SM, Wadsworth MH, Treacy D, Trombetta JJ, et al. Dissecting the multicellular ecosystem of metastatic melanoma by single-cell RNA-seq. Science (1979) [Internet]. 2016 Apr 8;352(6282):189–96. Available from: https://www.science.org/doi/10.1126/science.aad0501
4. Patel AP, Tirosh I, Trombetta JJ, Shalek AK, Gillespie SM, Wakimoto H, et al. Single-cell RNA-seq highlights intratumoral heterogeneity in primary glioblastoma. Science (1979) [Internet]. 2014 Jun 20;344(6190):1396–401. Available from: https://www.science.org/doi/10.1126/science.1254257
5. Kumar MP, Du J, Lagoudas G, Jiao Y, Sawyer A, Drummond DC, et al. Analysis of Single-Cell RNA-Seq Identifies Cell-Cell Communication Associated with Tumor Characteristics. Cell Rep. 2018 Nov;25(6):1458-1468.e4.
